# The tumor vasculature an attractive CAR T cell target in solid tumors

**DOI:** 10.1007/s10456-019-09687-9

**Published:** 2019-10-18

**Authors:** Parvin Akbari, Elisabeth J. M. Huijbers, Maria Themeli, Arjan W. Griffioen, Judy R. van Beijnum

**Affiliations:** 1 Angiogenesis Laboratory, Department of Medical Oncology, Cancer Center Amsterdam, Vrije Universiteit Amsterdam, Amsterdam, The Netherlands; 2 Department of Stem Cells and Regenerative Medicine, National Institute of Genetic Engineering and Biotechnology (NIGEB), Tehran, Iran; 3 Department of Hematology, Cancer Center Amsterdam, Amsterdam UMC, Vrije Universiteit Amsterdam, Amsterdam, The Netherlands

**Keywords:** CAR T cells, Vascular targeting, Angiogenesis, Immunotherapy

## Abstract

T cells armed with a chimeric antigen receptor, CAR T cells, have shown extraordinary activity against certain B lymphocyte malignancies, when targeted towards the CD19 B cell surface marker. These results have led to the regulatory approval of two CAR T cell approaches. Translation of this result to the solid tumor setting has been problematic until now. A number of differences between liquid and solid tumors are likely to cause this discrepancy. The main ones of these are undoubtedly the uncomplicated availability of the target cell within the blood compartment and the abundant expression of the target molecule on the cancerous cells in the case of hematological malignancies. Targets expressed by solid tumor cells are hard to engage due to the non-adhesive and abnormal vasculature, while conditions in the tumor microenvironment can be extremely immunosuppressive. Targets in the tumor vasculature are readily reachable by CAR T cells and reside outside the immunosuppressive tumor microenvironment. It is therefore hypothesized that targeting CAR T cells towards the tumor vasculature of solid tumors may share the excellent effects of CAR T cell therapy with that against hematological malignancies. A few reports have shown promising results. Suggestions are provided for further improvement.

Anti-angiogenic therapy has firmly entered the clinical management of cancer, but induction of long patient survivals with angiostatic- or vascular-targeted drugs is still a challenge [1]. Pharmaceutical strategies are mainly based on neutralizing tumor-produced growth factors and blocking their receptors with antibodies and kinase inhibitors. This strategy is not efficient and appears to be prone to drug-induced resistance, as the most prominent targets are the tumor cells themselves [2]. Therefore, a better strategy would be to directly target the vascular lining through specific markers of the tumor endothelium. Immunotherapies are continuously being developed and the revelation of the checkpoint inhibitors have finally led to long-term remissions and possibly cures. An immunotherapy strategy directly targeted at the tumor vasculature is therefore believed to be promising. A very interesting development is the use of engineered T lymphocytes equipped with chimeric antigen receptors with predefined specificity, referred to as CAR T cells [3]. This strategy has been extremely efficient when used for B cell acute lymphoblastic leukemia and when targeted against CD19, showing complete responses in > 80% of patients. This success is explained by the fact that infused CAR T cells engage leukemic cells within the vascular compartment and access to the molecular target is therefore extremely efficient. Using a CAR-based therapy against solid tumors has turned out to be much more challenging [4], since the first hurdle is active extravasation. This process is problematic in tumors as the angiogenic conditions induce dysfunctional vessels and endothelial cell anergy, resulting in a non-adhesive endothelial lining [5]. The next obstacle for a T cell to overcome is the strong anti-inflammatory microenvironment inside the tumor. Immune suppressive cytokines such as IL10, TGFβ and VEGF, regulatory T cells, and macrophages with a deviated maturation (myeloid-derived suppressor cells) are abundantly present in the tumor microenvironment, causing even aggressive activated anti-tumor cytotoxic T cells to become inactive.

Although the difficulty caused by the immune suppression in the tumor might be alleviated by co-treatment with checkpoint inhibitors, it is hypothesized that a CAR T cell strategy against the tumor vasculature will address both of the above-mentioned difficulties. On the one hand, the molecular target is readily accessible from within the vasculature and on the other, the effector cell does not need to enter the immune suppressive atmosphere. Several studies have been reported where CAR T cells were targeted to tumor vascular markers. A few of these were aiming for targeting VEGFR-2 [6, 7]. This approach was effective in several mouse models; however, the above-mentioned argument on resistance to the therapy applies to this target. A promising report was presented on targeting prostate specific membrane antigen (PSMA) [8]. This approach was shown to be successful in an ovarian cancer model and it was suggested that effects in patients where PSMA is also expressed by the tumor cells can be more pronounced [8]. Targeting tumor endothelial marker (TEM)-8 by CAR therapy was reported to treat triple-negative breast cancer (TNBC) [9]. This strategy resulted in a steeply decreasing tumor size immediately after the CAR T cell infusion. Although the treatment did not completely eradicate the TNBC xenografts, tumors were still significantly smaller and contained significantly less blood vessels after a period of 2 months [9]. An interesting approach was reported by Xie et al. [10], who directed a CAR therapy against the EIIIB domain containing fibronectin splice variant. This fibronectin isoform is expressed by tumor cells in many types of cancer, but has also been reported to appear in the angiogenic vasculature of tumors. This approach was successful in immunocompetent-, but not in immunodeficient mice, suggesting a role for endogenous immunity as well.

Although there is little evidence to date that CAR T cell therapies can become a valid treatment option for patients with solid tumors, there are a number of promising reports in recent literature. One of these is the concept of targeting CAR T cells towards the tumor vasculature. While promising activities have been demonstrated, none of the mentioned studies led to complete eradication of solid tumors. Next to general improvements of CAR T cells, such as optimization of CAR design [3] and introduction of homing and migration (chemokine) receptors or means to overcome metabolic barriers [4], opportunities for further improvement of CAR T cell approaches towards solid tumors involve the selection of target molecules that are more abundantly expressed in the tumor vasculature. It is suggested that vascular targets present at the tumor vasculature-matrix interface can especially be beneficial as disruption of the vascular integrity may cause enhanced bystander immunity [10]. In addition, selection of target molecules that have a more abundant expression at the luminal face of the tumor endothelium could increase engagement by, and therefore accessibility for, CAR T cells. Alternatively, the use of CAR T cells harboring CARs recognizing more than one target can enhance affinity, as well as specificity and therefore safety, of the engineered T cells towards the angiogenic vasculature.
